# Inhibitors of Oxidative Phosphorylation Modulate Astrocyte Inflammatory Responses through AMPK-Dependent Ptgs2 mRNA Stabilization

**DOI:** 10.3390/cells8101185

**Published:** 2019-10-01

**Authors:** Alina Astakhova, Dmitry Chistyakov, Dominique Thomas, Gerd Geisslinger, Bernhard Brüne, Marina Sergeeva, Dmitry Namgaladze

**Affiliations:** 1 Belozersky Institute of Physico-Chemical Biology, Moscow State University, 119992 Moscow, Russia; alina_astakhova@yahoo.com (A.A.); chistyakof@gmail.com (D.C.); mg.sergeeva@gmail.com (M.S.); 2 Pharmazentrum Frankfurt, Institute of Clinical Pharmacology, Faculty of Medicine, Goethe-University Frankfurt, 60590 Frankfurt, Germany; Thomas@med.uni-frankfurt.de (D.T.); geisslinger@em.uni-frankfurt.de (G.G.); 3 Fraunhofer Institute for Molecular Biology and Applied Ecology IME, Branch for Translational Medicine and Pharmacology TMP, 60596 Frankfurt, Germany; 4 Institute of Biochemistry I, Faculty of Medicine, Goethe-University Frankfurt, 60590 Frankfurt, Germany; B.Bruene@biochem.uni-frankfurt.de

**Keywords:** astrocytes, inflammation, mitochondria, prostaglandins

## Abstract

Inflammatory activation of astroglia adds to the pathology of various neurological diseases. Astrocytes respond to microglia-derived cytokines such as interleukin-1α (IL-1α) with enhanced inflammatory signaling. This provokes pro-inflammatory gene expression of, among others, the eicosanoid-generating enzyme prostaglandin endoperoxide synthase 2 (Ptgs2). Whereas metabolic regulation of innate immune cell inflammatory responses is intensely studied, pathways related to how metabolism modulates inflammatory signaling in astrocytes are underexplored. Here, we examined how mitochondrial oxidative phosphorylation affects inflammatory responses towards IL-1α and tumor necrosis factor α in neonatal rat astrocytes. Blocking respiratory complex I and III or adenosine triphosphate (ATP) synthase did not affect activation of inflammatory signaling by IL-1α, but did elicit differential effects on inflammatory gene mRNA expression. Remarkably, mRNA and protein expression of Ptgs2 by IL-1α was consistently up-regulated when oxidative phosphorylation was inhibited. The increase of Ptgs2 resulted from mRNA stabilization. Mitochondrial inhibitors also increased IL-1α-triggered secretion of eicosanoids, such as prostaglandin E_2_, prostaglandin F_2α_, and 6-keto-prostaglandin F_1α_, as assessed by liquid chromatography/mass spectrometry. Mechanistically, attenuating oxidative phosphorylation elevated adenosine monophosphate (AMP) and activated AMP-activated protein kinase (AMPK). AMPK silencing prevented Ptgs2 up-regulation by mitochondrial inhibitors, while AMPK activators recapitulated Ptgs2 mRNA stability regulation. Our data indicate modulation of astrocyte inflammatory responses by oxidative metabolism, with relevance towards eicosanoid production.

## 1. Introduction

Inflammatory processes within the central nervous system (CNS) underlie the pathology of neurologic disorders such as multiple sclerosis, and contribute to other neurodegenerative diseases, including Alzheimer’s disease, Parkinson’s disease, and amyotrophic lateral sclerosis [1]. Whereas cells of the mononuclear phagocyte system, in particular brain-resident microglia, are major perpetuators of neuroinflammation, the contribution of other CNS-resident cells is being increasingly recognized. Astrocytes are CNS-resident cells with important homeostatic functions, but which also have roles in the pathogenesis of neurologic disorders [2]. In response to pro-inflammatory cytokines released by activated microglia, in particular interleukin (IL)-1α and tumor necrosis factor-α (TNFα), astrocytes acquire an activated, neurotoxic phenotype (A1 polarization) [3,4] and upregulate pro-inflammatory genes, which may be influenced by environmental insults [5]. In addition to typical pro-inflammatory and neurotoxic genes, part of the gene expression program may be devoted to resolution of inflammation, with gene products serving context-dependent pro-inflammatory or pro-resolving functions [6]. A prominent example is the inducible prostaglandin endoperoxide synthase (Ptgs2), a key enzyme of eicosanoid biosynthesis. Its products, prostaglandins, show both pro-inflammatory as well as pro-resolving qualities [6]. Our recent studies revealed complex regulation of Ptgs2 expression at both the transcriptional and post-transcriptional levels in primary rat astrocytes [7,8,9].

Cellular metabolism controls inflammatory gene expression in cells of the innate and adaptive immune systems [10,11]. Although glycolytic metabolism has established roles in pro-inflammatory immune cell activation [10], mitochondrial activity significantly contributes to regulation of immune responses through a variety of mechanisms [12]. Astrocytes are considered to rely mostly on glycolytic metabolism, providing lactate to fuel neuronal oxidative metabolism [13]. However, astrocytes also display robust mitochondrial activity and are able to consume different metabolic substrates, including fatty acids and glutamate [14,15]. How mitochondria in astrocytes regulate inflammatory responses is poorly understood.

We evaluated how mitochondrial activity regulates inflammatory gene expression in primary rat cortical astrocytes. Whereas mitochondrial activity is dispensable for the induction of several inflammatory genes, some genes, including Ptgs2, are modulated. Mechanistically, deficient mitochondrial adenosine triphosphate (ATP) generation triggers adenosine monophosphate (AMP)-activated protein kinase (AMPK)-dependent Ptgs2 mRNA stabilization, resulting in increased prostanoid generation.

## 2. Materials and Methods

### 2.1. Cell Culture and Treatments

Cultures of primary rat astrocytes were obtained from newborn rats of both sexes. In brief, brains from decapitated newborn rats were dissected into PBS on ice, and re-suspended in 0.25% Trypsin- ethylenediaminetetraacetic acid (EDTA) followed by incubation at 37 °C for 1 hour. Cells were dissociated using a 100 µm cell strainer, plated into 175 cm^2^ culture flasks in Dulbecco’s modified Eagle’s medium (DMEM) containing 10% fetal calf serum and 100 μg/mL penicillin and streptomycin, followed by culture at 37 °C, 5% CO_2_. After 5 days of cultivation in DMEM, microglia cells were removed by shaking for 4 hours at 300 rpm. After an additional 5–7 days, cells were re-plated into 6-well or 12-well plates and maintained for 2 days in DMEM before use. Cells were treated for indicated times with 5 ng/ml interleukin-1α (IL-1α) (400-01A, Peprotech, Hamburg, Germany), 20 ng/ml tumor necrosis factor-α (TNFα) (400-14, Peprotech, Hamburg, Germany), 1 µM rotenone (13995, Cayman, Ann Arbor, MI, USA), 2.5 µM oligomycin (11342, Cayman, Ann Arbor, MI, USA), 10 µg/ml antimycin A (A8674, Sigma-Aldrich, Taufkirchen, Germany), 250 µM phenformin (P7045, Sigma-Aldrich, Taufkirchen, Germany), 100 µM A-769662 (3336, Tocris, Minneapolis, MN, USA ), 5 µg/ml actinomycin D (A4262, Sigma-Aldrich, Taufkirchen, Germany), or 100 µM mitoTEMPO (16621, Cayman, Ann Arbor, MI, USA).

### 2.2. RNA Isolation and Analysis

Total RNA from astrocytes was isolated using PeqGold RNAPure kit (PeqLab, Erlangen, Germany) and reverse transcribed using Maxima First Strand cDNA Synthesis kit (Thermo Scientific, Waltham, MA, USA). Quantitative real-time polymerase chain reaction (PCR) was carried out on a CFX96 system from Bio-Rad using iQ SYBR^TM^ green (Bio-Rad, Hercules, CA, USA). Primer sequences are available upon request. Target gene expression was normalized to the expression of actin.

### 2.3. Western Blotting

Total cell lysates were prepared by scraping the cells into Laemmli buffer (2% sodium dodecyl sulfate (SDS), 62.5 mM Tris-HCl, pH 6.8, 10% glycerol, 10 mM dithiothreitol (DTT)) containing protease inhibitors (Complete, Roche, Basel, Switzerland) followed by sonification, centrifugation at 10000g for 10 minutes, and heating at 95 °C for 5 minutes. Protein lysates were run on 7.5% polyacrylamide gels and blotted on nitrocellulose membranes. The following primary antibodies were used: Ptgs2 (#12282), phospho-IκB kinase (pIKK) (S176/S180) (#2697), IκB-kinase β (IKKβ) (#8943) phospho-cJun N-terminal kinase (pJNK) (T183/Y185) (#4668), cJun N-terminal kinase (JNK) (#9252), phospho-p38(T180/Y182) (#4511), p38 (#9212), phospho-AMPK (T172) (#2535), AMPKα1 (#2795), phospho-acetyl-CoA carboxylase (pACC) (S79) (#3661), acetyl-CoA carboxylase (ACC) (#3676) (all Cell Signaling Technology, Frankfurt, Germany), HuR (sc-5261, Santa-Cruz, Heidelberg, Germany), and tubulin (T4026, Sigma-Aldrich, Taufkirchen, Germany). Membranes were incubated with IRDye 700/800-coupled secondary antibodies, and scanned and quantified using an Odyssey imaging system (Licor, Bad Homburg, Germany).

### 2.4. siRNA Transfection

Silencing of rat Prkaa1 was performed using siGENOME SMARTpool (Dharmacon, Lafayette, CO, USA) at 50 nM and Hyperfect transfection reagent (Qiagen, Hilden, Germany). Cells were treated 96 hours post-transfection.

### 2.5. ATP Determination

ATP levels in cellular lysates were determined using CellTiter-Glo kit (Promega, Mannheim, Germany) according to manufacturer instructions.

### 2.6. AMP Determination by LC-MS

The concentrations of AMP, ADP, and ATP in the samples were analyzed by liquid chromatography-electrospray ionization-tandem mass spectrometry, as previously described [16]. Briefly, methanolic cell extracts (approximately 750 µl samples) were spiked with 20 µl internal standard solution (^13^C_10_,^15^N_5_-AMP and ^13^C_10_,^15^N_5_-ATP, 100 ng/ml each in methanol), centrifuged, and the clear supernatant was evaporated at 45 °C under a gentle stream of nitrogen. Adenosine phosphates were separated using an anion exchange high-performance liquid chromatography (HPLC) column (BioBasic AX, 150x2.1 mm, Thermo Fisher, Waltham, MA, USA) within 15 minutes. A mass spectrometer 5500 QTrap (Sciex, Darmstadt, Germany) was used as an analyzer, operating as triple quadrupole in positive multiple reaction monitoring mode. The analysis of ATP was performed as previously described [16]. Additionally, AMP and ADP were quantified using ^13^C_10_,^15^N_5_-AMP as internal standard. The precursor-to-product ion transitions used as quantifiers were m/z 348.2 → 136.1 for AMP and m/z 427.8 → 136.1 for ADP. Data acquisition and quantification were performed using Analyst software version 1.6.3 and MultiQuant software version 3.0.2 (both Sciex, Darmstadt, Germany), respectively, employing the internal standard method (isotope dilution mass spectrometry). Calibration curves were calculated by linear regression with 1/x weighting.

### 2.7. Lipidome Analysis by Ultra Performance Liquid Chromatography—Tandem Mass Spectrometry (UPLC-MS/MS)

Cell-free culture media (0.5 ml) were mixed with 2 ng of deuterated internal standard solutions, centrifuged (12000× *g*, 3 min), mixed with 6 ml of 0.1% acetic acid, and loaded onto Oasis® PRiME hydrophilic lipophilic balance (HLB) solid-phase lipid extraction cartridge (60 mg, 3cc, (Waters, Milford, MA, USA) catalogue number 186008056). The cartridge was washed with 2 ml of 15% methanol containing 0.1% formic acid and the lipids were sequentially eluted with 500 μl of anhydrous methanol and 500 μl of acetonitrile. The resulting samples were mixed, concentrated by evaporation of the solvent under gentle stream of nitrogen, and stored at −80 °C. The target eicosanoids were identified by triple quadrupole UPLC-MS/MS (Shimadzu 8040, Kyoto, Japan) under previously reported run conditions [17] and quantified by comparing their MS, MS/MS, and UPLC (retention times, peak areas) data with the data obtained for deuterated internal standard compounds (6-keto PGF_1α_-d4 (cat.no. 315210), TXB_2_-d4 (cat.no. 319030), PGF_2α_-d4 (cat.no. 316010), PGE_2_-d4 (cat.no. 314010), PGD_2_-d4 (cat.no. 312010), LTB_4_-d4 (cat.no. 320110), 12(S)-HETE-d8 (cat.no. 334570) (Cayman Chemical, Ann Arbor, MI, USA) of the same classes using Lipid Mediator Version 2 software package (Shimadzu, Kyoto, Japan).

### 2.8. Statistical Analysis

Data are presented as means ± SE of at least three independent experiments. Normality of data sets was tested using Shapiro–Wilk test. Data were analyzed by one-way ANOVA analysis of variance with Bonferroni post hoc means comparison using GraphPad Prism version 5.03, GraphPad, San Diego, CA, USA). Differences were considered statistically significant at *p* < 0.05.

## 3. Results

**Figure 1:**
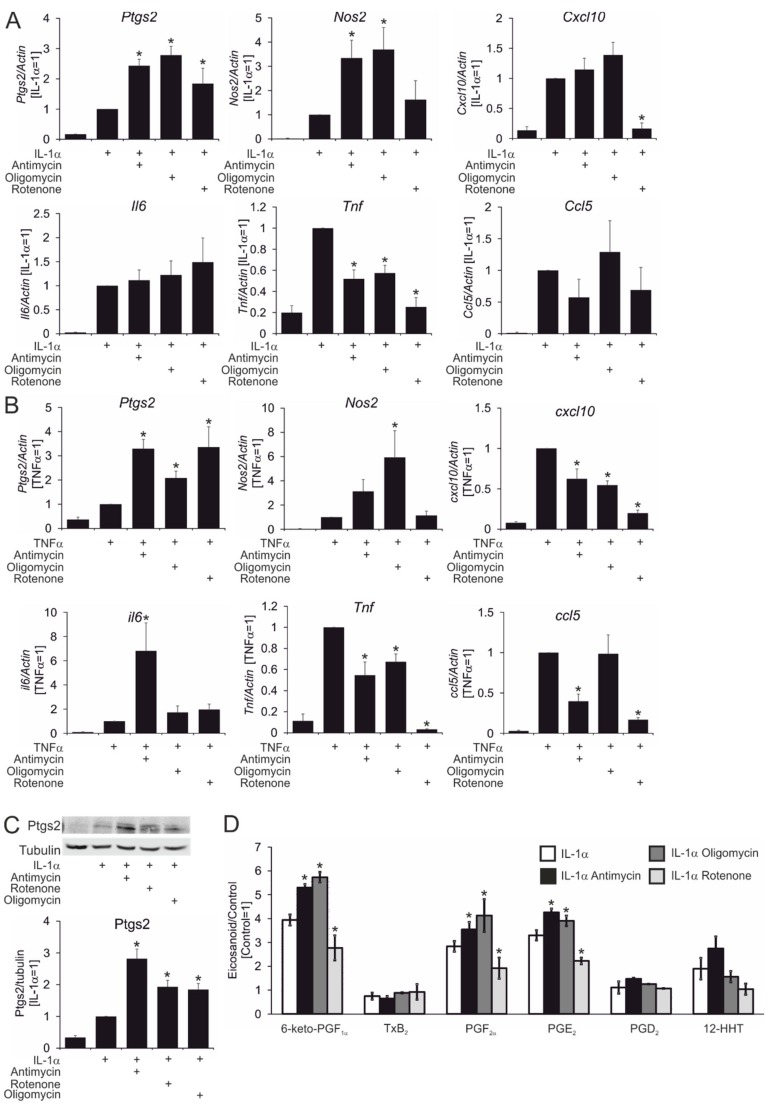
Inhibitors of mitochondrial activity increase prostaglandin endoperoxide synthase 2 (Ptgs2) expression in rat cortical astrocytes exposed to pro-inflammatory cytokines. (**A**,**B**) The mRNA expression of indicated genes in astrocytes treated for 3 hours with interleukin-1α (IL-1α )(**A**) or with tumor necrosis factor-α (TNF-α) (**B**); in both cases, cells were also treated with antimycin, oligomycin, or rotenone, as indicated. (**C**) Western blot analysis of Ptgs2 expression in astrocytes treated for 4 hours with IL-1α in the presence or absence of rotenone, oligomycin, and antimycin, as indicated. (**D**) Comparative lipidomic analysis. Concentrations of eicosanoids in supernatants of astrocytes treated for 4 hours with IL-1α in the presence or absence of rotenone, oligomycin, and antimycin, and measured using ultra performance liquid chromatography - tandem mass spectrometry. Note: *, *p* < 0.05 vs. IL-1α or TNFα. Data represent mean values ± SE of 3 or 4 independent experiments.

Whereas the ability of astrocytes to respond to pro-inflammatory stimuli with increased mRNA expression of various pro-inflammatory genes is well documented, the question of how astrocyte metabolism affects these responses has not been addressed so far. To analyze the impact of mitochondrial respiration on inflammatory responses of astrocytes, we exposed primary rat cortical astrocytes to pro-inflammatory stimuli typically released by activated microglia (TNFα and IL-1α) [4] for 3 h. This was done in the absence or presence of inhibitors of mitochondrial respiratory complexes I (rotenone) and III (antimycin), as well as mitochondrial ATP synthase (oligomycin), followed by analysis of mRNA expression of genes associated with pro-inflammatory astrocyte polarization. Preliminary experiments verified that the inhibitors, at concentrations used in this study, suppressed oxygen consumption in astrocytes. As seen in Figure 1A, IL-1α robustly induced the expression of typical pro-inflammatory mRNAs. We observed heterogeneous modulation of IL1α-induced mRNA expression by mitochondrial inhibitors. Whereas antimycin and oligomycin did not influence the induction of *Cxcl10*, *Ccl5*, and *Il6* mRNAs, they decreased IL-1α-stimulated levels of *Tnf* mRNA, and increased mRNA expression of *Ptgs2*, as well as inducible nitric oxide synthase (*Nos2*). We noticed that all inhibitors affected *Ptgs2* expression to a similar extent, while rotenone had a strong inhibitory effect on *Tnf* and *Cxcl10* expression but failed to potentiate IL-1α-induced *Nos2* mRNA expression. Divergent effects of mitochondrial inhibitors were observed using TNFα as a stimulus (Figure 1B). Whereas *Ptgs2*, *Tnf*, and *Nos2* expression followed the same pattern of dependency as for IL-1α stimulation, expression of *Cxcl10* and *Ccl5* was suppressed by inhibitors of oxidative phosphorylation (except for the lack of the effect of oligomycin on *Ccl5*). *Il6* induction remained intact in the presence of rotenone and oligomycin, but was potentiated by antimycin. Together, these data suggest that the influence of mitochondrial inhibitors on inflammatory mRNA expression is stimulus- and gene-dependent. In further experiments, we focused on the effect of mitochondrial inhibitors on *Ptgs2* expression, since it was up-regulated by all inhibitors of oxidative phosphorylation, regardless of stimulus. Next, we confirmed the potentiating effect of mitochondrial inhibitors on the expression of Ptgs2 at the protein level (Figure 1C). Furthermore, several prostaglandin downstream products of the Ptgs2 enzymatic activity accumulated in cell supernatants of IL-1α-treated astrocytes after co-incubation with rotenone, antimycin, and oligomycin (Figure 1D). PGH_2_, which is generated upon activation of Ptgs2, is metabolized by various secondary enzymes into eicosanoids with different biological activities [18]. Therefore, we estimated the eicosanoid spectrum using UPLC-MS/MS. Several metabolites of the cyclooxygenase pathway were detectable in the culture medium of control and IL1α-stimulated cells: 6-keto-PGF_1α_ (a stable derivative of PGI_2_), PGF_2α_, PGE_2_, PGD_2_, TXB_2_ (a stable derivative of TxA_2_), and 12-HHT (12-Hydroxyheptadecatrienoic acid), which may be produced from PGH_2_ through thromboxane synthase-dependent and -independent pathways [19]. IL-1α did not alter levels of TXB_2_ and PGD_2_, but markedly induced the release of 6-keto-PGF_1α_, PGF_2α_, PGE_2_, and 12-HHT (Figure 1D). In accordance with their effects on Ptgs2 protein (Figure 1C), antimycin and oligomycin potentiated IL-1α-stimulated release of 6-keto-PGF_1α_, PGF_2α_, and PGE_2_, but rotenone suppressed secretion of these eicosanoids, indicating that rotenone may inhibit cyclooxygenase activity. 

**Figure 2:**
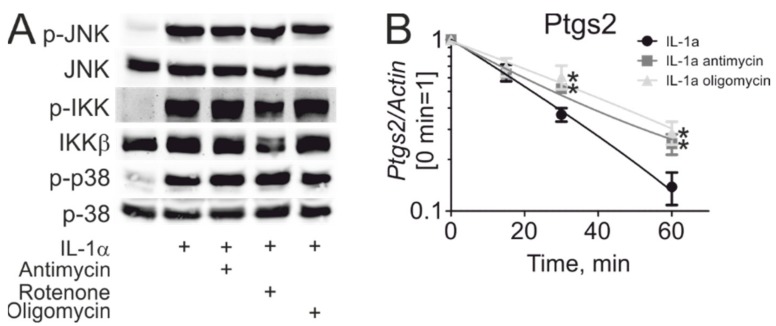
Inhibitors of oxidative phosphorylation increase *Ptgs2* mRNA stability. (**A**) Western blot analysis of c-Jun N-terminal kinase (JNK), IκB kinase (IKK), and p38 mitogen-activated protein kinase (MAPK) phosphorylation in astrocytes treated for 0.5 hours with IL-1α in the presence or absence of rotenone, oligomycin, and antimycin, as indicated. (**B**) The mRNA expression of Ptgs2 in astrocytes treated with IL-1α, antimycin, and oligomycin for 1 hour followed by actinomycin D for indicated times. Note: *, *p* < 0.05 vs. IL-1α. Data represent mean values ± SE of 3 or 4 independent experiments.

Next, we addressed mechanisms of how mitochondrial inhibitors potentiate *Ptgs2* mRNA expression. Analyzing early signaling by IL-1α, we found no effect of mitochondrial inhibitors on activation of the nuclear factor κB (NFκB) signaling (phosphorylation of IκB kinase (IKK)α/β), or the activities of c-Jun N-terminal kinase (JNK) and p38 mitogen-activated protein kinase (MAPK) (Figure 2A), suggesting that early pro-inflammatory signaling cascades underlying cytokine-induced transcriptional activation do not require mitochondrial activity. Since levels of *Ptgs2* mRNA are regulated both by transcriptional and RNA stability mechanisms, we then assessed stability of *Ptgs2* mRNA by analyzing its half-life in IL-1α-treated cells. Therefore, we pre-treated astrocytes with IL-1α in the presence or absence of antimycin and oligomycin, followed by the addition of the transcription inhibitor actinomycin D. In actinomycin-treated cells, the levels of *Ptgs2* mRNA rapidly decay, a reflection of the low stability of *Ptgs2* mRNA. Interestingly, mitochondrial inhibitors delayed the decay of *Ptgs2* transcripts, indicating that increased *Ptgs2* mRNA stability may underlie the stimulatory effect of mitochondrial inhibitors on *Ptgs2* gene expression (Figure 2B).

**Figure 3:**
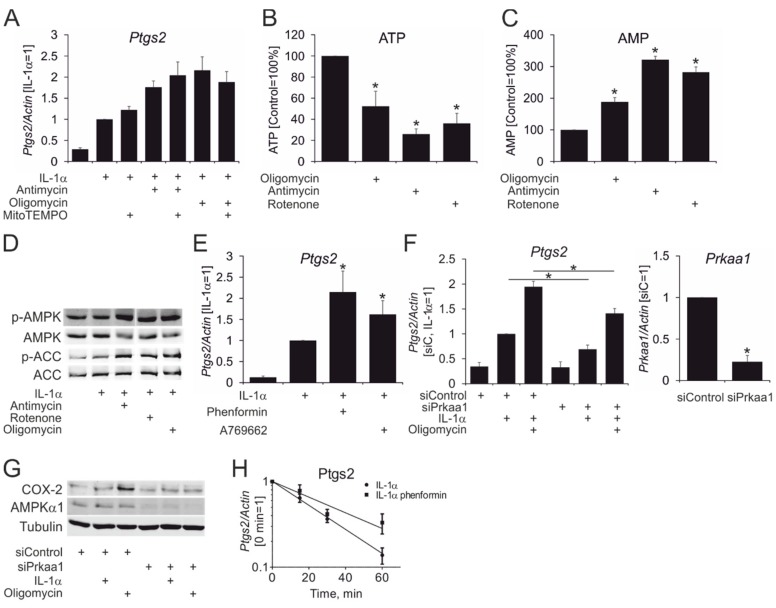
Role of adenosine monophosphate (AMP)-activated protein kinase (AMPK) in potentiating Ptgs2 expression by mitochondrial inhibitors. (**A**) The mRNA expression of *Ptgs2* in astrocytes treated with IL-1α for 3 hours in the absence or presence of mito-TEMPO, antimycin, and oligomycin, as indicated. (**B**,**C**) Intracellular adenosine triphosphate (ATP) (**B**) and AMP (**C**) levels in astrocytes treated with rotenone, antimycin, and oligomycin for 1 hour. (**D**) Western blot analysis of AMPK and acetyl-CoA carboxylase (ACC) phosphorylation in astrocytes treated for 1 hour with IL-1α in the absence or presence of rotenone, oligomycin, and antimycin, as indicated. (**E**) *Ptgs2* mRNA expression in astrocytes treated with IL-1α, phenformin, and A-769662 for 3 hours. (**F**) The mRNA expression of *Ptgs2* and *Prkaa1* in astrocytes transfected with control or Prkaa1 siRNAs for 96 hours and treated for 3 hours with IL-1α in the presence or absence of oligomycin as indicated. (**G**) Western blot analysis of Ptgs2 and Prkaa1 expression in astrocytes transfected with control or Prkaa1 siRNAs for 96 hours and treated for 4 hours with IL-1α in the presence or absence of oligomycin, as indicated. (**H**) The mRNA expression of *Ptgs2* in astrocytes treated with IL-1α and phenformin for 1 hour followed by actinomycin D for indicated times. Note: *, *p* < 0.05 vs. untreated (**B**,**C**), IL-1α (**E**), or vs. siControl for each treatment condition (**F**). Data represent mean values ± SE of 3 or 4 independent experiments.

We then questioned how mitochondrial inhibition causes mRNA stabilization. Respiratory inhibition decreased ATP production, thus, increasing the AMP/ATP ratio and activating the major energy sensor AMP-activated protein kinase (AMPK). At the same time, mitochondrial inhibition may also increase levels of mitochondrial reactive oxygen species (ROS), thereby eliciting ROS-dependent signaling. However, the mitochondrial ROS blocker mito-TEMPO did not influence IL-1α-stimulated *Ptgs2* mRNA expression in the presence or absence of antimycin or oligomycin (Figure 3A). On the other hand, mitochondrial inhibitors reduced cellular ATP content (Figure 3B) and increased AMP concentration in cell lysates, as assessed by LC-MS (Figure 3C). Expectedly, these alterations activated AMPK, as assessed by increased phosphorylation of AMPK and its target acetyl-CoA carboxylase (ACC) (Figure 3D). Next, we explored pharmacological AMPK activation towards *Ptgs2* gene expression in IL-1α-treated astrocytes. As seen in Figure 3E, the pharmacological AMPK activators phenformin and A-769662 potentiated IL-1α-induced mRNA expression of *Ptgs2*. To confirm the role of AMPK in potentiation of IL-1α-elicited *Ptgs2* expression by mitochondrial inhibitors, we silenced expression of the predominant α1-isoform of the catalytic AMPK subunit (Prkaa1). As shown in Figure 3F,G, Prkaa1 silencing effectively suppressed *Prkaa1* mRNA and protein expression, and largely attenuated *Ptgs2* mRNA and protein in astrocytes treated with IL-1α and oligomycin. Finally, we observed increased mRNA stability of *Ptgs2* in the presence of the AMPK activator phenformin (Figure 3H). Taken together, our data suggest that the potentiating effect of mitochondrial inhibitors on cytokine-stimulated *Ptgs2* gene expression in astrocytes is AMPK-mediated.

## 4. Discussion

The novel finding of this study is a gene-specific susceptibility of the inflammatory response of primary rat astrocytes to inhibition of mitochondrial oxidative phosphorylation. Using several pharmacological inhibitors targeting different components of the mitochondrial respiratory chain, we show that cytosolic kinase signaling cascades induced by pro-inflammatory cytokines remain largely intact in the absence of functional oxidative phosphorylation. This may reflect the reliance of astrocytes on glycolytic metabolism [13]. Similarly, inhibitors of oxidative phosphorylation do not generally suppress inflammatory responses of professional phagocytes [20], showing nuanced regulation of selected cytokine responses dependent on e.g. ROS generation by individual respiratory complexes [21,22]. Interestingly, our data also show cell-type-specific differences of individual cytokine induction depending on intact respiratory chains. In contrast to observations of macrophages where increased Tnf expression in response to TLR agonists was insensitive to mitochondrial inhibitors [22,23], in astrocytes, all drugs targeting oxidative phosphorylation (especially complex I inhibitor rotenone) attenuated cytokine-induced *Tnf* mRNA. We also noticed stimulus-dependent effects of mitochondrial inhibitors on the expression of chemokines *Cxcl10* and *Ccl5*, with only TNFα-induced expression being sensitive. The causes of these discrepant effects remain to be investigated. 

Mechanistically, we observed an AMPK-dependent *Ptgs2* mRNA stabilization to drive increased Ptgs2 expression and activity in cytokine-treated astrocytes upon inhibition of oxidative phosphorylation. Although some reports suggested ROS-dependent AMPK activation due to redox modification of AMPK [24,25], our data support a classical model of AMPK activation due to an elevated AMP/ATP ratio in astrocytes treated with mitochondrial inhibitors, consistent with other detailed studies showing that ROS-dependent activation of AMPK proceeds through alteration of cellular AMP/ATP [26,27]. Remarkably, despite being considered a glycolytic cell type, astrocytes display substantial ATP losses upon inhibition of oxidative phosphorylation. This may be explained by the reliance of the first enzyme of glycolytic cascade, hexokinase, on mitochondria-derived ATP, facilitated by the mitochondrial location of hexokinase in neurons and astroglia [28,29]. Therefore, astrocytes may not be able to rapidly compensate for the mitochondrial ATP production loss by rapid upregulation of glycolysis. We note that although inhibiting mitochondrial ROS did not alter *Ptgs2* upregulation by inhibitors of oxidative phosphorylation, expression of other inflammatory genes, such as IL-1β, is known to undergo regulation by mitochondrial ROS, as well as by mitochondrial membrane potential in innate immune cells [21]. Furthermore, AMPK activation following mitochondrial dysfunction is known to cause mitochondrial fragmentation [30], which also may contribute to altering inflammatory gene expression, as we have recently shown [31]. Whether these mechanisms contribute to modulating inflammatory gene expression in astrocytes remains to be addressed in future research. It should also be explored whether *Ptgs2* induction by other cytokines present in inflammatory milieu of activated microglia, such as IL-6, is similarly affected by inhibiting mitochondrial activity

How does AMPK stabilize *Ptgs2* mRNA? Several proteins are involved in the regulation of *Ptgs2* mRNA stability [32]. Most prominent are the RNA-binding proteins HuR (stabilizing) [33] and tristetraprolin (TTP) (destabilizing) [34]. HuR is an attractive candidate AMPK target, since AMPK has been reported to increase cytoplasmic levels of HuR through phosphorylation of nuclear importin [35]. In contrast, *Ptgs2* stability may be negatively affected by AMPK due to TTP up-regulation by mTORC1 signaling [36], considering that AMPK is a well-established inhibitor of mTORC1 [37,38]. Although our previous research showed the relevance of TTP and HuR regulation for inflammatory gene regulation in astrocytes [39], we could not find evidence for altered cytosolic levels of HuR or TTP after mitochondrial inhibition in our system (data not shown). At least 16 proteins may bind *Ptgs2* 3’- untranslated region [32]. Furthermore, deregulated expression of miRNAs may contribute to altered stability of Ptgs2 transcript under our conditions. Considering this complexity, the link between AMPK and Ptgs2 stability surely warrants further research.

An important finding of our work is that mitochondrial activity differentially affects the spectra of synthesized eicosanoids. The functions of astrocytes are sensitive to modulation by various eicosanoids [17]. In this work, we observed that astrocytes responded to stimulation by IL-1α released 6-keto-PGF_1α_, PGF_2α_, PGE_2_, and 12-HHT, but not PGD_2_ and TXB_2_. This is different from astrocyte responses to stimulation by LPS, where increased production of PGE_2_, TXB_2_, 6-keto-PGF_1α_, and PGD_2_ was documented [40]. Apparently, stimulus-dependent effects were operating on the arachidonic acid metabolism system. Traditionally, changes in the expression of Ptgs correlate well with changes in the level of secreted PGE_2_, since Ptgs is considered to be a limiting enzyme in the bienzyme system for eicosanoid synthesis from arachidonic acid. Our data on the spectrum of eicosanoids during stimulation with IL-1α and modulation by mitochondrial inhibition indicate a currently unobserved relationship between mitochondrial activity and the enzymes of downstream terminal eicosanoid synthesis. This opens the possibility of changing the ratio between eicosanoids, not only through the influence on the enzymes of their synthesis, but also through the regulation of mitochondrial activity. We also noticed that rotenone exerted differential effects, attenuating secretion of multiple prostanoids while increasing Ptgs2 expression. These observations indicate additional effects due either to complex I inhibition or to other off-target actions of rotenone, either suppressing Ptgs2 enzymatic activity or inhibiting the delivery of the Ptgs2 substrate, arachidonic acid. This may also be specific to cell culture conditions, as rotenone did not affect kidney PGE_2_ levels when applied in a whole animal model [41].

In conclusion, our work shows that mitochondria contribute to inflammatory responses of astroglial cells. Apparently, AMPK activation through energy loss is only one of several mitochondrial-dependent mechanisms to regulate inflammatory gene expression. Whereas *Ptgs2* expression in neuronal and glial cells is usually connected to increased pain, we previously connected increased *Ptgs2* stability to an anti-inflammatory spectrum of activities of the PPARγ-activating drug rosiglitazone [9]. Whether documented activation of AMPK by rosiglitazone contributes to this effect remains an interesting, unexplored possibility [42].

## References

[1] Peruzzotti-Jametti L., Pluchino S. (2018). Targeting Mitochondrial Metabolism in Neuroinflammation: Towards a Therapy for Progressive Multiple Sclerosis. Trends Mol. Med..

[2] Rothhammer V., Quintana F.J. (2015). Control of autoimmune CNS inflammation by astrocytes. Semin. Immunopathol..

[3] Liddelow S.A., Barres B.A. (2017). Reactive Astrocytes: Production, Function, and Therapeutic Potential. Immunity.

[4] Liddelow S.A., Guttenplan K.A., Clarke L.E., Bennett F.C., Bohlen C.J., Schirmer L., Bennett M.L., Münch A.E., Chung W.-S., Peterson T.C. (2017). Neurotoxic reactive astrocytes are induced by activated microglia. Nature.

[5] Wheeler M.A., Jaronen M., Covacu R., Zandee S.E.J., Scalisi G., Rothhammer V., Tjon E.C., Chao C.-C., Kenison J.E., Blain M. (2019). Environmental Control of Astrocyte Pathogenic Activities in CNS Inflammation. Cell.

[6] Gilroy D.W., Bishop-Bailey D. (2019). Lipid mediators in immune regulation and resolution. Br. J. Pharmacol..

[7] Aleshin S., Grabeklis S., Hanck T., Sergeeva M., Reiser G. (2009). Peroxisome proliferator-activated receptor (PPAR)-gamma positively controls and PPARalpha negatively controls cyclooxygenase-2 expression in rat brain astrocytes through a convergence on PPARbeta/delta via mutual control of PPAR expression levels. Mol. Pharmacol..

[8] Astakhova A.A., Chistyakov D.V., Pankevich E.V., Sergeeva M.G. (2015). Regulation of cyclooxygenase 2 expression by agonists of PPAR nuclear receptors in the model of endotoxin tolerance in astrocytes. Biochemistry Mosc..

[9] Pankevich E.V., Astakhova A.A., Chistyakov D.V., Sergeeva M.G. (2017). Antiinflammatory Effect of Rosiglitazone via Modulation of mRNA Stability of Interleukin 10 and Cyclooxygenase 2 in Astrocytes. Biochemistry Mosc..

[10] O’Neill L.A., Kishton R.J., Rathmell J. (2016). A guide to immunometabolism for immunologists. Nat. Rev. Immunol..

[11] Buck M.D., Sowell R.T., Kaech S.M., Pearce E.L. (2017). Metabolic Instruction of Immunity. Cell.

[12] Mehta M.M., Weinberg S.E., Chandel N.S. (2017). Mitochondrial control of immunity: Beyond ATP. Nat. Rev. Immunol..

[13] Magistretti P.J., Allaman I. (2018). Lactate in the brain: From metabolic end-product to signalling molecule. Nat. Rev. Neurosci..

[14] Eraso-Pichot A., Brasó-Vives M., Golbano A., Menacho C., Claro E., Galea E., Masgrau R. GSEA of mouse and human mitochondriomes reveals fatty acid oxidation in astrocytes. Glia.

[15] Dienel G.A. (2019). Brain Glucose Metabolism: Integration of Energetics with Function. Physiol. Rev..

[16] Thomas D., Herold N., Keppler O.T., Geisslinger G., Ferreirós N. (2015). Quantitation of endogenous nucleoside triphosphates and nucleosides in human cells by liquid chromatography tandem mass spectrometry. Anal. Bioanal. Chem..

[17] Chistyakov D.V., Grabeklis S., Goriainov S.V., Chistyakov V.V., Sergeeva M.G., Reiser G. (2018). Astrocytes synthesize primary and cyclopentenone prostaglandins that are negative regulators of their proliferation. Biochem. Biophys. Res. Commun..

[18] Gabbs M., Leng S., Devassy J.G., Monirujjaman M., Aukema H.M. (2015). Advances in Our Understanding of Oxylipins Derived from Dietary PUFAs. Adv. Nutr..

[19] Matsunobu T., Okuno T., Yokoyama C., Yokomizo T. (2013). Thromboxane A synthase-independent production of 12-hydroxyheptadecatrienoic acid, a BLT2 ligand. J. Lipid Res..

[20] Kelly B., Tannahill G.M., Murphy M.P., O’Neill L.A. (2015). Metformin Inhibits the Production of Reactive Oxygen Species from NADH:Ubiquinone Oxidoreductase to Limit Induction of Interleukin-1β (IL-1β) and Boosts Interleukin-10 (IL-10) in Lipopolysaccharide (LPS)-activated Macrophages. J. Biol. Chem..

[21] Mills E.L., Kelly B., Logan A., Costa A.S.H., Varma M., Bryant C.E., Tourlomousis P., Däbritz J., Henry M., Gottlieb E. (2016). Succinate Dehydrogenase Supports Metabolic Repurposing of Mitochondria to Drive Inflammatory Macrophages. Cell.

[22] Garaude J., Acín-Pérez R., Martínez-Cano S., Enamorado M., Ugolini M., Nistal-Villán E., Hervás-Stubbs S., Pelegrín P., Sander L.E., Enríquez J.A. (2016). Mitochondrial respiratory-chain adaptations in macrophages contribute to antibacterial host defense. Nat. Immunol..

[23] Tannahill G.M., Curtis A.M., Adamik J., Palsson-McDermott E.M., McGettrick A.F., Goel G., Frezza C., Bernard N.J., Kelly B., Foley N.H. (2013). Succinate is an inflammatory signal that induces IL-1β through HIF-1α. Nature.

[24] Zmijewski J.W., Banerjee S., Bae H., Friggeri A., Lazarowski E.R., Abraham E. (2010). Exposure to hydrogen peroxide induces oxidation and activation of AMP-activated protein kinase. J. Biol. Chem..

[25] Emerling B.M., Weinberg F., Snyder C., Burgess Z., Mutlu G.M., Viollet B., Budinger G.R.S., Chandel N.S. (2009). Hypoxic activation of AMPK is dependent on mitochondrial ROS but independent of an increase in AMP/ATP ratio. Free Radic. Biol. Med..

[26] Auciello F.R., Ross F.A., Ikematsu N., Hardie D.G. (2014). Oxidative stress activates AMPK in cultured cells primarily by increasing cellular AMP and/or ADP. FEBS Lett..

[27] Hinchy E.C., Gruszczyk A.V., Willows R., Navaratnam N., Hall A.R., Bates G., Bright T.P., Krieg T., Carling D., Murphy M.P. (2018). Mitochondria-derived ROS activate AMP-activated protein kinase (AMPK) indirectly. J. Biol. Chem..

[28] Rosa J.C., César M.d.C. (2016). Role of Hexokinase and VDAC in Neurological Disorders. Curr. Mol. Pharmacol..

[29] Kao-Jen J., Wilson J.E. (1980). Localization of hexokinase in neural tissue: Electron microscopic studies of rat cerebellar cortex. J. Neurochem..

[30] Toyama E.Q., Herzig S., Courchet J., Lewis T.L., Losón O.C., Hellberg K., Young N.P., Chen H., Polleux F., Chan D.C. (2016). Metabolism. AMP-activated protein kinase mediates mitochondrial fission in response to energy stress. Science.

[31] Zezina E., Snodgrass R.G., Schreiber Y., Zukunft S., Schürmann C., Heringdorf D.M.Z., Geisslinger G., Fleming I., Brandes R.P., Brüne B. (2018). Mitochondrial fragmentation in human macrophages attenuates palmitate-induced inflammatory responses. Biochim. Biophys. Acta Mol. Cell Biol. Lipids.

[32] Moore A.E., Young L.E., Dixon D.A. (2011). MicroRNA and AU-rich element regulation of prostaglandin synthesis. Cancer Metastasis Rev..

[33] Cok S.J., Acton S.J., Morrison A.R. (2003). The proximal region of the 3′-untranslated region of cyclooxygenase-2 is recognized by a multimeric protein complex containing HuR, TIA-1, TIAR, and the heterogeneous nuclear ribonucleoprotein U. J. Biol. Chem..

[34] Sawaoka H., Dixon D.A., Oates J.A., Boutaud O. (2003). Tristetraprolin binds to the 3′-untranslated region of cyclooxygenase-2 mRNA. A polyadenylation variant in a cancer cell line lacks the binding site. J. Biol. Chem..

[35] Wang W., Yang X., Kawai T., López de Silanes I., Mazan-Mamczarz K., Chen P., Chook Y.M., Quensel C., Köhler M., Gorospe M. (2004). AMP-activated protein kinase-regulated phosphorylation and acetylation of importin alpha1: Involvement in the nuclear import of RNA-binding protein HuR. J. Biol. Chem..

[36] Bayeva M., Khechaduri A., Puig S., Chang H.-C., Patial S., Blackshear P.J., Ardehali H. (2012). mTOR regulates cellular iron homeostasis through tristetraprolin. Cell Metab..

[37] Gwinn D.M., Shackelford D.B., Egan D.F., Mihaylova M.M., Mery A., Vasquez D.S., Turk B.E., Shaw R.J. (2008). AMPK phosphorylation of raptor mediates a metabolic checkpoint. Mol. Cell.

[38] Inoki K., Ouyang H., Zhu T., Lindvall C., Wang Y., Zhang X., Yang Q., Bennett C., Harada Y., Stankunas K. (2006). TSC2 integrates Wnt and energy signals via a coordinated phosphorylation by AMPK and GSK3 to regulate cell growth. Cell.

[39] Astakhova A.A., Chistyakov D.V., Sergeeva M.G., Reiser G. (2018). Regulation of the ARE-binding proteins, TTP (tristetraprolin) and HuR (human antigen R), in inflammatory response in astrocytes. Neurochem. Int..

[40] Chistyakov D.V., Azbukina N.V., Astakhova A.A., Goriainov S.V., Chistyakov V.V., Sergeeva M.G. (2018). Sex-Mediated Differences in LPS Induced Alterations of TNFα, IL-10 Expression, and Prostaglandin Synthesis in Primary Astrocytes. Int. J. Mol. Sci..

[41] Zhang Y., Sun Y., Ding G., Huang S., Zhang A., Jia Z. (2015). Inhibition of Mitochondrial Complex-1 Prevents the Downregulation of NKCC2 and ENaCα in Obstructive Kidney Disease. Sci. Rep..

[42] Fryer L.G., Parbu-Patel A., Carling D. (2002). The Anti-diabetic drugs rosiglitazone and metformin stimulate AMP-activated protein kinase through distinct signaling pathways. J. Biol. Chem..

